# The Archaeal Transcription Termination Factor aCPSF1 is a Robust Phylogenetic Marker for Archaeal Taxonomy

**DOI:** 10.1128/spectrum.01539-21

**Published:** 2021-12-08

**Authors:** Jie Li, Xiaowei Zheng, Lingyan Li, Shengjie Zhang, Mifang Ren, Li Huang, Xiuzhu Dong

**Affiliations:** a State Key Laboratory of Microbial Resources, Institute of Microbiology, Chinese Academy of Sciences, Beijing, China; b College of Life Science, University of Chinese Academy of Sciences, Beijing, China; Dublin City University

**Keywords:** archaeal taxonomy, aCPSF1, phylogenetic marker, phylogenomics, transcription termination factor

## Abstract

Archaea are highly diverse and represent a primary life domain, but the majority of them remain uncultured. Currently, 16S rRNA phylogeny is widely used in archaeal taxonomy and diversity surveys. However, highly conserved sequence of 16S rRNA possibly results in generation of chimera in the amplicons and metagenome-assembled genomes (MAGs) and therefore limits its application. The newly developed phylogenomic approach has overcome these flaws, but it demands high-quality MAGs and intensive computation. In this study, we investigated the use of the archaeal transcription termination factor aCPSF1 in archaeal classification and diversity surveys. The phylogenetic analysis of 1,964 aCPSF1 orthologs retrieved from the available archaeal (meta)genomes resulted in convergent clustering patterns with those of archaeal phylogenomics and 16S rRNA phylogeny. The aCPSF1 phylogeny also displayed comparable clustering with the methanoarchaeal McrABG phylogeny and the haloarchaeal phylogenomics. Normalization of 779 aCPSF1 sequences including 261 from cultured archaeal species yielded a taxonomic ranking system with higher resolutions than that obtained with 16S rRNA for genus and species. Using the aCPSF1 taxonomy, 144 unclassified archaea in NCBI database were identified to various taxonomic ranks. Moreover, aCPSF1- and 16S rRNA-based surveys of the archaeal diversity in a sample from a South China Sea cold seep produced similar results. Our results demonstrate that aCPSF1 is an alternative archaeal phylogenetic marker, which exhibits higher resolution than 16S rRNA, and is more readily usable than phylogenomics in the taxonomic study of archaea.

**IMPORTANCE** Archaea represent a unique type of prokaryote, which inhabit in various environments including extreme environments, and so define the boundary of biosphere, and play pivotal ecological roles, particularly in extreme environments. Since their discovery over 40 years ago, environmental archaea have been widely investigated using the 16S rRNA sequence comparison, and the recently developed phylogenomic approach because the majority of archaea are recalcitrant to laboratory cultivation. However, the highly conserved sequence of 16S rRNA and intensive bioinformatic computation of phylogenomics limit their applications in archaeal species delineation and diversity investigations. aCPSF1 is a ubiquitously distributed and vertically inherited transcription termination factor in archaea. In this study, we developed an aCPSF1-based archaeal taxonomic system which exhibits congruent phylogenic clustering patterns with archaeal phylogenomics and higher resolution than 16S rRNA in distinguishing archaea at lower taxonomic ranks. Therefore, aCPSF1 is a new phylogenetic marker in the taxonomic and diversity studies of archaea.

## INTRODUCTION

*Archaea*, in parallel with *Bacteria* and *Eukaryotes*, represent the third domain of cellular life and comprise highly diverse prokaryotic phyla. They are ubiquitously distributed in every corner of the earth, such as soils, oceans, and particularly in extreme environments and, therefore, are believed to play significant roles in biogeochemical recycling of carbon, nitrogen, and other elements. Since the domain *Archaea* was proposed by Carl Woese in 1977 based on the sequence analysis of the small subunit rRNA genes (1), extensive surveys based on 16S rRNA sequence analyses have been carried out to understand the archaeal diversity and abundance in almost every region of Earth (2–7), because most archaeal species are recalcitrant to be cultivated.

Currently, 16S rRNA gene is the phylogenetic marker primarily used in prokaryote taxonomy, identification of cultured species, and surveys of prokaryote diversity. To data, millions of 16S rRNA gene sequences have been compiled in quality-controlled public databases like NCBI, SILVA, and others (8–11), and therefore, this molecule serves as a robust tool in rapid identification of bacterial or archaeal isolates. However, the highly conserved sequence of the 16S rRNA genes in prokaryotes results in a very low phylogenetic resolution of 98.7% or even 99.5% sequence identity among species, and the full-length sequences are required for accurate identification of higher taxa (12). Additionally, the high sequence similarity of the 16S rRNA gene is prone to produce chimera amplicons in the diversity surveys of environmental species, which artificially inflates diversity estimations and introduces noise into phylogenetic trees (13–16). To alleviate these problems, a variety of alternative genes, including the housekeeping genes in single or in concatenation, or specific gene categories, have been used in phylogenetic analysis of various archaeal groups. For instance, the DNA gyrase gene is used in the taxonomic study of Sulfolobaceae (17), a concatenation of 32 conserved genes are used in Haloarchaea (18), and *mcrA*, the methyl coenzyme M reductase A subunit gene, has been applied in the phylogenetic and diversity study of methanogenic archaea (19, 20). The concatenated marker protein trees derived from isolated and population genomes are much less susceptible to chimeric artifacts (16, 21).

In recent years, the substantial development of culture-independent metagenome sequencing techniques has provided unprecedent access to the metagenomes of most uncultured archaeal lineages. Metagenome information not only opens an avenue to explore the diversity and metabolic potentials, but also lays a foundation for phylogenomic studies of the largely uncultured archaeal species, which has resulted in a rapid growth of the archaeal phylogenetic tree with a number of new phyla, classes and orders (22–24). Very recently, Parks et al. (25, 26) have developed a Genome Taxonomy Database (GTDB), the first comprehensive prokaryote taxonomy based on phylogenomic analysis, which inferred phylogenetic trees from the concatenation of single-copy vertically inherited genes, and provided higher resolution than those based on single gene. The GTDB has also recorded amazingly diverse archaea, and more than 40 phyla are proposed, however, with the majority encompassing only a few or even no cultured species (26).

MAG facilitated phylogenomics provides a robust approach capable of affiliating and identifying unknown prokaryotic species to their phylogenetic placements without culturing. However, it is labor-intensive and costly to obtain a high-quality MAG and demands highly intensive computation to identify an unknown species based on the concatenation of about one hundred of proteins. For example, 3,840 computational hours were required to construct a maximum likelihood tree based on the concatenation of 16 conserved ribosomal proteins from 3,083 genomes (27). Meanwhile, 16S rRNA genes are frequently filtrated out during MAG assembling due to the conserved sequences among species, thus precluding the use of 16S rRNA as a single marker in identifying or exploring new archaeal taxa from environmental sequences.

Recently, we reported that the aCPSF1 protein functions as a general transcriptional termination factor of archaea (28). aCPSF1 is an endonuclease affiliated with the β-CASP RNase family, and the encoding gene is ubiquitous in the sequenced archaeal genomes (29). It was found that the phylogeny of the aCPSF1 orthologs from 110 selected archaeal complete genomes exhibited a similar clustering topology to that of the concatenation of 53 archaeal ribosomal proteins (29), suggesting that aCPSF1 could be used as an archaeal phylogenetic marker. In this work, we retrieved 2,520 archaeal genomes/MAGs from the public databases (up to February 2020), and constructed aCPSF1- and 16S rRNA-based phylogenetic trees as well as a phylogenomic tree based on a concatenation of 122 conserved archaeal proteins. Remarkably, convergent clustering patterns were found among the three trees, indicating that aCPSF1 is an alternative phylogenetic marker of archaea. Sequence identity normalization of 779 aCPSF1 proteins, including 261 from cultured strains with taxonomic placements, reveals a distinguishable six-taxonomic rank from species to phylum, which particularly exhibits higher resolutions on the lower taxonomic ranks of archaea, i.e., on genus and species. Therefore, the archaeal conserved aCPSF1 can be an alternative phylogenetic marker which has higher resolution than 16S rRNA and is more efficient of computational time than phylogenomic analysis in archaeal taxonomic study.

## RESULTS AND DISCUSSION

### The aCPSF1 orthologs are ubiquitously distributed in the genomes/MAGs of archaea.

Phung et al. reported that the aCPSF1 orthologs are highly conserved in 110 selected archaeal complete genomes (29). The recently developed metagenomic sequencing approach has generated a wealth of genomic data of uncultured and unidentified archaeal lineages. In this study, we re-analyzed the distribution and conservation of the aCPSF1 orthologs based on the expanded archaeal genomes/MAGs data. Through sequence alignment to the β-CASP RNase aCPSF1 (TIGR03675) using hmmsearch (30), a total of 4,860,490 archaeal protein were retrieved and searched for the aCPSF1 orthologs from 2,520 archaeal (meta)genomes (Dataset S1). The resultant 2,060 aCPSF1 orthologs are derived from 1,964 genomes, and most genomes (1,873/1,964, 95.4%) contain a single copy of the aCPSF1 gene, except that some haloarchaea (56/1,964, 2.9%) and unclassified archaea (35/1,964, 1.8%) possess two and occasionally three copies per genome (Dataset S1).

**FIG 1:**
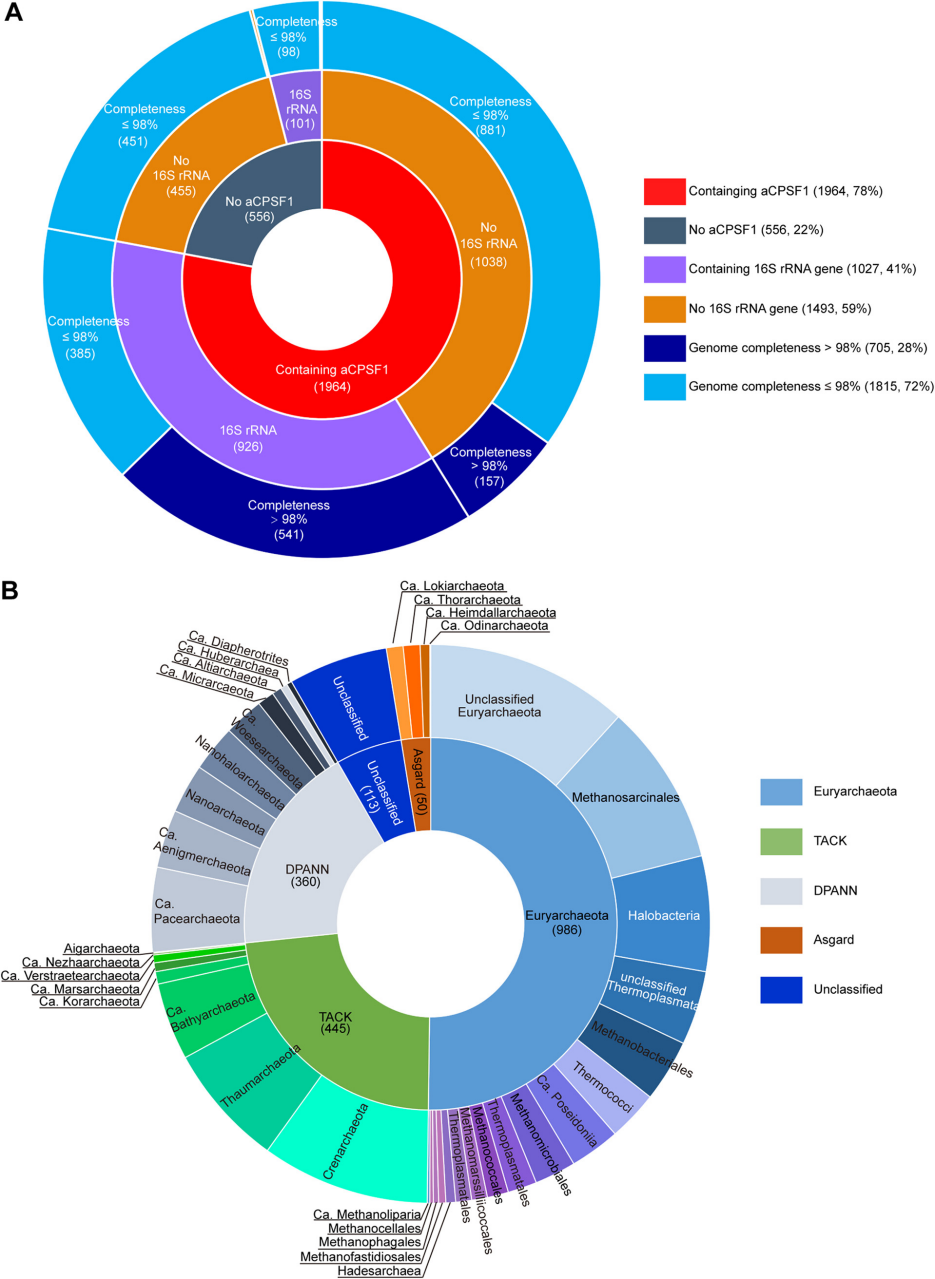
Statistics of the aCPSF1 orthologs in the (meta)genomes deposited in the genome databases and its distribution in various archaeal phyla. (A) The pie chart shows the statistics of the aCPSF1orthologs and 16S rRNA gene distributed in the 2,520 available archaeal genomes/MAGs deposited in NCBI and GTDB databases and the genome quality. The outer ring indicates the numbers of genomes that have a completeness >98% or not; the middle ring shows the genome numbers that contain 16S rRNA gene(s); and the inner ring indicates the genome numbers that contain an aCPSF1 gene ortholog. (B) The pie chart shows the phylogenetic distribution of 1,964 aCPSF1 orthologs retrieved in this study. The inner ring shows the percentages of aCPSF1 distribution among the four superphyla of archaea, and the outer ring indicates those among various phyla or classes.

In all, aCPSF1 orthologs are found in 78% of the archaeal genomes/MAGs (1,964/2,520), and the remaining genomes/MAGs (556/2,520) except for one are less than 98% complete (Fig. 1A). In comparison, the 16S rRNA genes are missing in 59% of the retrieved genomes/MAGs (1,493/2,520), even some of which are greater than 98% complete (6.2%, 157/2,520), presumably because the high sequence similarity of 16S rRNAs among species prevents them from being assembled MAGs (16). As shown in Fig. 1B, the retrieved aCPSF1-carrying genomes/MAGs encompass all defined archaeal phyla and orders, and include cultured and enriched representatives. Approximately half of the aCPSF1 orthologs are from *Euryarchaeota*, most likely because more cultivated archaea are classified in this phylum. The above data indicate that aCPSF1 orthologs are widely distributed among archaeal phyla and, therefore, can be employed in the phylogenetic analysis and taxonomic study of archaea.

### The archaeal phylogenetic tree based on the aCPSF1 protein exhibits similar clustering topology with those based on the 16S rRNA gene and the phylogenomics.

First, aCPSF1 orthologs from 42 representative species that encompass all defined archaeal phyla were analyzed for amino acid sequence similarity. Sequence alignment shows that the aCPSF1 orthologs are markedly conserved over nearly the entire length (Fig. S1), including the two N-terminal K homolog (KH) domains, the central MβL domain, and C-terminal β-CASP domain, and the seven conserved motifs, which are featured in β-CASP ribonucleases of the metallo-β-lactamase superfamily (29, 31, 32), thereby confirming that the retrieved genes encode aCPSF1 orthologs.

**FIG 2:**
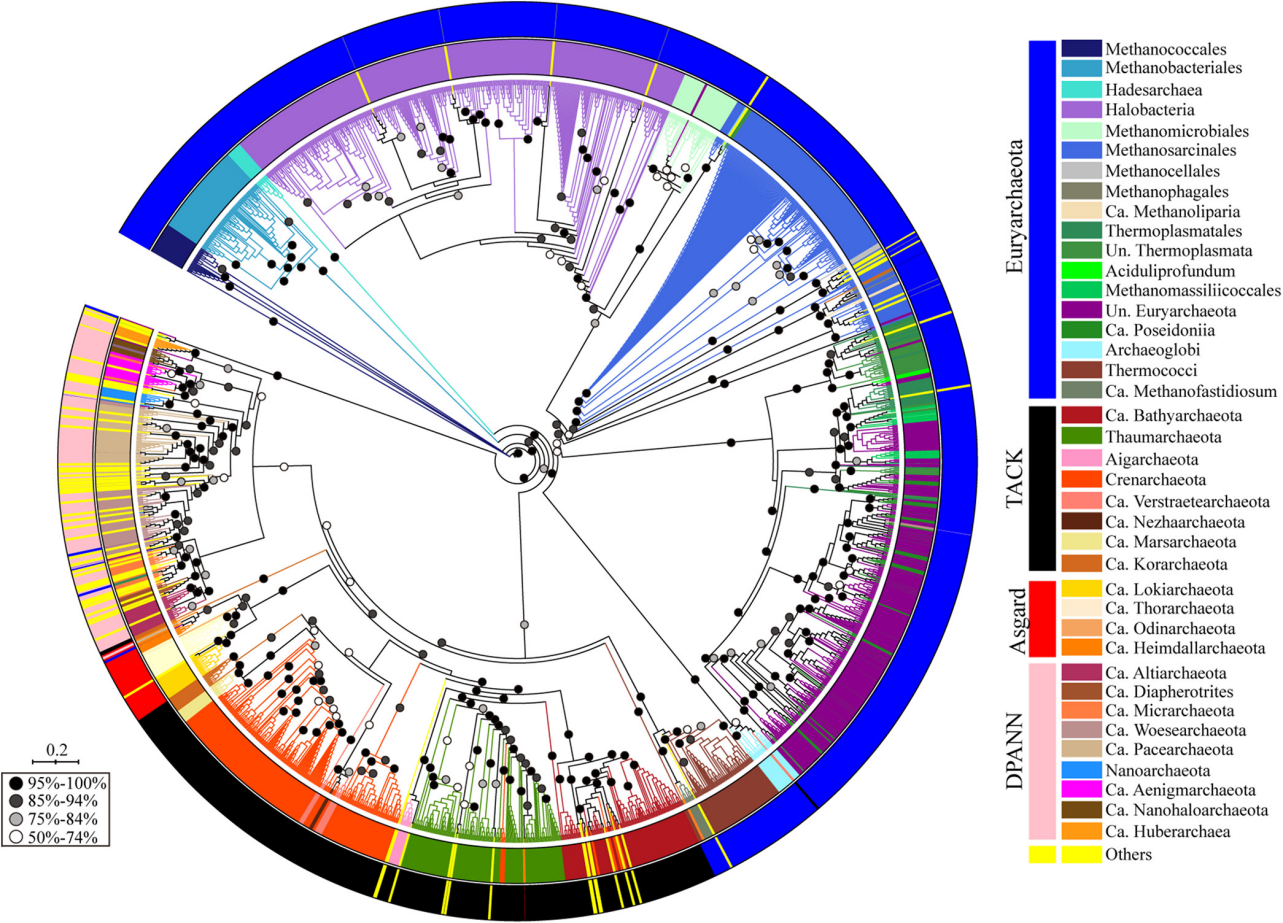
Phylogenetic analysis of the aCPSF1 orthologs from 1,964 archaeal genomes/MAGs. In total, 1,964 aCPSF1 orthologs were retrieved from 2,520 available archaeal genomes/MAGs deposited in NCBI database. These genomes all have protein annotation information and phylogenetic affiliations. Un, unclassified; Ca, Candidatus; Others, unclassified and uncultured archaea. A maximum likelihood phylogenetic tree was constructed based on consensus amino acids of aCPSF1 protein sequences, inferred with FastTree v.2.1.10 under the WAG+GAMMA model (IQ-TREE 1.6.12 in the LG+C20+R4+F model, 1,000 ultrafast bootstraps replicates [52, 57, 58]), and visualized using iTOL v3 (59). Bootstrap supporting values of branch clustering are indicated by dots. Scale bar indicates number of substitutions per site.

Next, we used 1,964 aCPSF1 proteins (Dataset S1) by each representative per genome to construct the archaeal phylogenetic tree. As shown in Fig. 2, a congruent taxonomic clustering pattern with that of concatenated 122 archaeal marker proteins (20, 23) was found, i.e., most of the aCPSF1 orthologs from the same superphyla or phyla (subgroups) were clustered in a similar fashion as that of phylogenomics. This suggests that the aCPSF1 orthologs could have emerged prior to the divergence of the lineages and been inherited vertically along with the species evolution in archaea, and so this conserved protein could be a candidate for a good phylogenetic marker in archaeal taxonomy. Additionally, an obvious computational time advantage was noticed on aCPSF1-based taxonomic clustering analysis as that construction of the 1,964 archaeal aCPSF1 phylogenetic tree with 1,000 bootstrap replicates using IQ-TREE (v.1.6.12) spent 360 computational hours in total, however, at least 42-fold more computational hours spent for the phylogenomic tree of 122 concatenated archaeal marker proteins from the same set of genomes on our computation platform.

**FIG 3:**
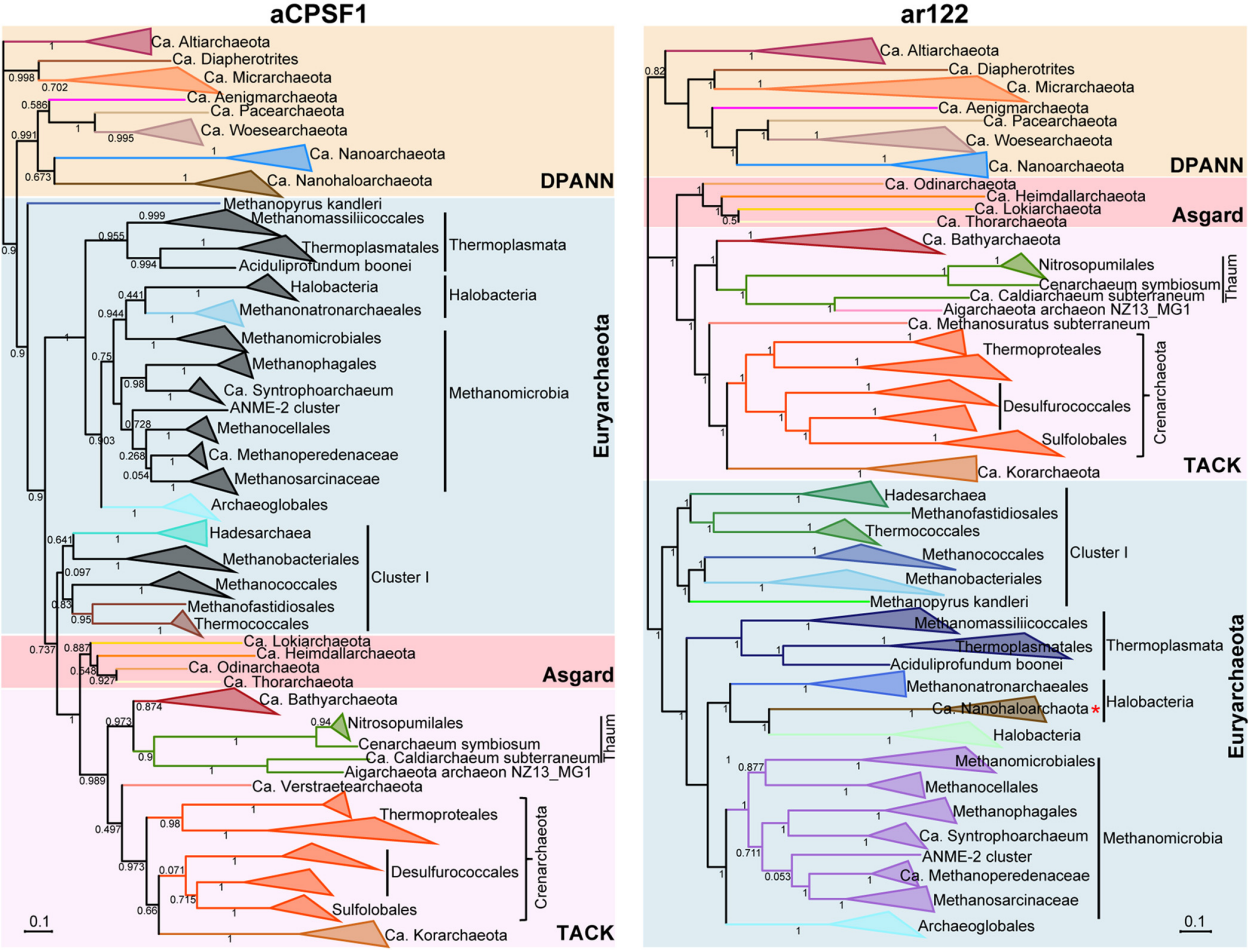
Comparison of the aCPSF1 phylogenetic topology with the phylogenomic clustering of representative *Archaea*. The aCPSF1 orthologs and 122 archaeal marker proteins were retrieved from the same 143 representative cultured archaeal genomes or completed MAGs with high quality. Consensus sequences of the aCPSF1 proteins (left) and the concatenated 122 archaeal proteins (right) were used to construct the respective maximum likelihood phylogenetic trees using IQ-TREE (v.1.6.12) with “LG+I+G4” mode and 1,000 times ultrafast. The phylogenic trees were visualized with iTOL v3 (https://itol.embl.de/). Bootstrap evaluation values of 1,000 iterations are indicated at branch nodes. Scale bar indicates substitution numbers per site.

To further analyze the applicability of aCPSF1 as a phylogenetic marker, aCPSF1 orthologs, concatenation of 122 marker proteins and 16S rRNA genes were chosen from 143 representatives that are derived from the four archaeal superphyla (Euryarchaeota, TACK, Asgard, and DPANN) and are mostly cultured or have complete high-quality genomes (Dataset S1, marked with “†”) for phylogenetic analysis. It was found that the phylogenetic trees generated using the three taxonomic systems display very similar clustering topology (Fig. 3 and Fig. S2), and the tested archaeal taxa were all clustered as three major clans: DPANN, *Euryarchaeota*, and TACK/Asgard, in which the Asgard archaea were consistently clustered with the TACK superphylum either as a root or parallel outgroup. Noteworthily, most archaeal clades above families in the aCPSF1 phylogenetic tree were respectively grouped in similar pattern as those in phylogenomic and 16S rRNA trees. However, the DPANN clan-hood, a deep-branching superphylum encompassing the nanosized archaea with reduced genomes (33, 34), and especially the phyla *Nanoarchaeota* and *Nanohaloarchaeota*, exhibited varying phylogenetic placements in the three phylogenies in this study, while similar varying placements of these (super)phyla were also noticed in the previous phylogenies (20, 23, 24, 33, 35).

Collectively, the aCPSF1 phylogeny in general generates the same phyla clustering pattern as those of the phylogenomics and 16S rRNA phylogenies of archaea. Given its highly conserved sequence, ubiquitous distribution among archaea and existence as a single copy gene in most genomes, aCPSF1 can be an alternative phylogenetic marker used in classification and diversity investigation of archaea and identification of the unknown taxa.

### Phylogenies of the methanoarchaeal aCPSF1 and the methyl-CoM reductase subunits McrABG concatenation display similar clustering patterns.

In the aCPSF1 phylogeny, the euryarchaeal subclades were coincidently grouped into two clusters with high bootstrapping support as the previously defined Cluster I and Cluster II *Euryarchaea* (22), except for very low bootstrap support of grouping the two families of *Methanoperedenaceae* and *Methanosarcinaceae*. To further investigate the applicability of this phylogenetic marker in the taxonomy of methanogenic archaea—the archaeal group containing the most cultured species and producing ample methane, we compared the aCPSF1 phylogeny with that of concatenated McrABG that comprises the Methyl-coenzyme M reductase complex (MCR). McrABG are the methanogenic and methanotrophic archaea signature proteins that catalyze methyl-coenzyme M reduction to methane or the reverse reaction (20). Remarkably, these proteins exhibit 61%–69% amino acid sequence similarities among the cultured methanogens. Concatenation of McrABG has been widely employed in the phylogenetic study and diversity surveys of methanogenic and methanotrophic archaea, revealing not only the underestimated diversity of methanogenic archaea in environment (19, 20, 36) but also non-methanogenic archaea carrying methanogenesis marker genes. These non-methanogenic archaea are affiliated with *Crenarchaeota* and *Bathyarchaeota,* the remote relatives of the conventional methanogenic and methanotrophic archaea in *Euryarchaeota* (37, 38).

**FIG 4:**
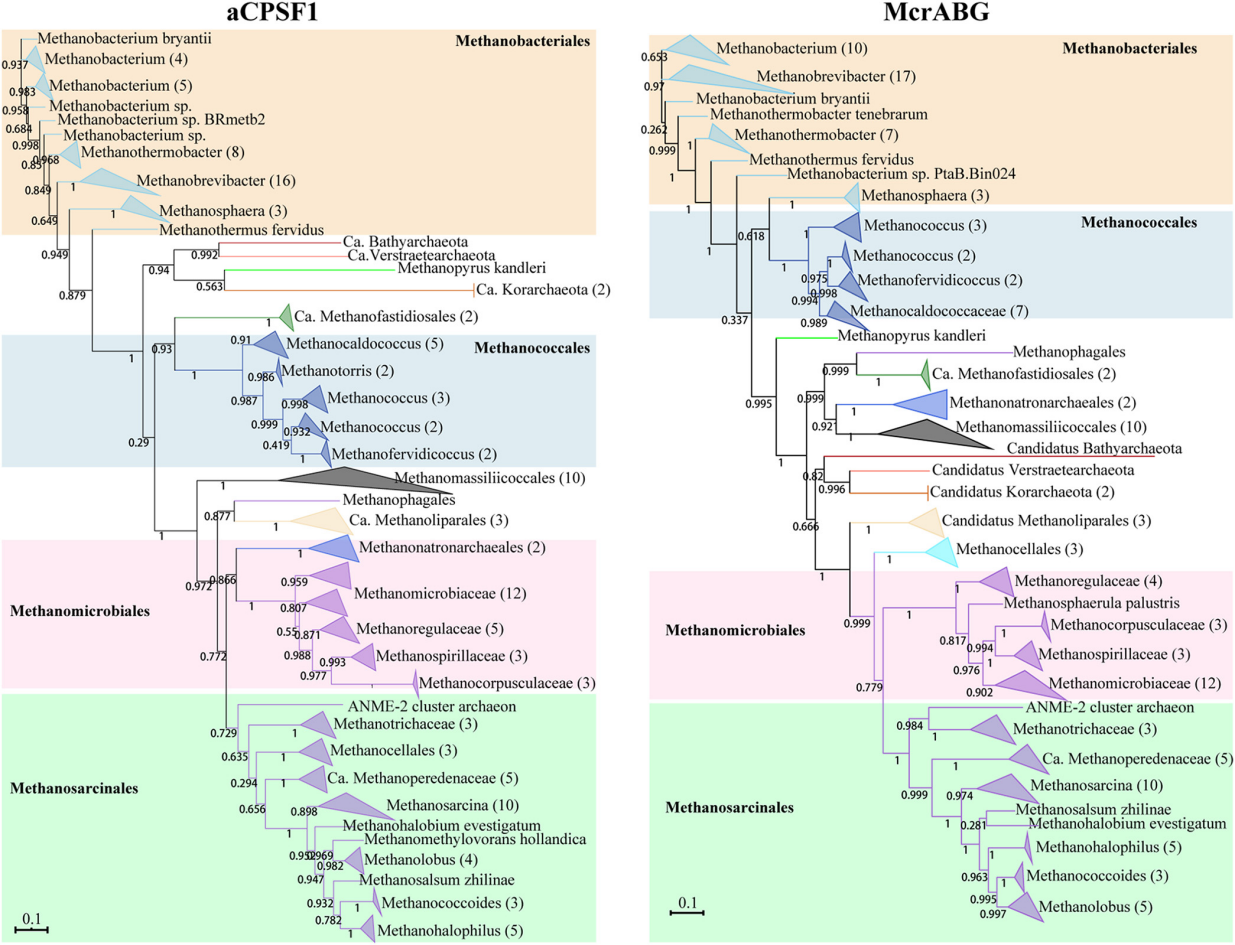
Comparison of the phylogenetic trees of methanogenic archaea constructed based on aCPSF1 orthologs and the McrABG concatenation. In total, 138 methanoarchaeal genomes listed in Dataset S1 (“*” marked) representing all seven defined methanogenic orders and methanotrophic archaea were selected, and the aCPSF1 and McrABG orthologs were retrieved for phylogenetic study. The maximum likelihood phylogenetic trees were respectively constructed based on the protein sequences of aCPSF1 orthologs (left) and McrABG protein concatenation (right). Inside the parenthesis following the methanogens indicate the genome numbers from the same taxa. Bootstrap evaluation values of 1,000 iterations are indicated at branch nodes. Scale bar indicates number of substitutions per site.

We selected aCPSF1 orthologs and McrABG proteins from the same 138 genomes of methanogenic/methanotrophic archaea (Dataset S1, “*” marked) for phylogenic study. These genomes all represent the identified methanogenic orders and newly found methanotrophic archaea that encode McrABG-like complexes, such as *Bathyarchaeota*. Phylogenetic analysis of the methanogenic and methanotrophic aCPSF1 orthologs resulted in a general congruent clustering pattern as that of the McrABG protein concatenation (Fig. 4). In the two phylogenies, the six orders that are defined based on 16S rRNA phylogeny were respectively clustered, namely, *Methanomicrobiales*, *Methanobacteriales*, *Methanococcales*, *Methanosarcinales*, *Methanomassiliicoccales*, and *Methanopyrales*, and in addition to newly defined orders: *Methanonatronarchaeales*, and candidatus orders of *Methanoliparales*, *Methanofastidiosales*, and *Methanophagales*. The only divergent clustered pattern is that the order *Methanocellales*, which is grouped within the order *Methanosarcinale*s in the aCPSF1 phylogeny. The hyperthermophile *Methanopyrales* as well as the non-euryarchaeal “methanogens” from TACK superphylum were consistently clustered as separate clades in both the aCPSF1 and McrABG phylogenies, consistent with the fact that the methyl-coenzyme M reductase-like protein complex carried by the TACK archaea differs from the authentic MCR. Because of the low similarity between the McrABG of methanogens and TACK archaea, the methanogenic markers in TACK were unlikely to be acquired via horizontal gene transfer.

Nevertheless, a few of the newly defined orders were differently clustered in the aCPSF1 phylogeny and the McrABG tree. For example, the candidatus order *Methanoliparales* was grouped as a separated clade in the McrABG tree, whereas it was clustered with the candidatus order *Methanophagales* in the aCPSF1 phylogeny. Clustering of the candidatus order *Methanoliparales* with anaerobic methane oxidizers (ANME) *Methanophagales* (ANME-1) may reveal a close phylogenetic relationship between the two that carry equivalent metabolic potentials, with the former comprising anaerobes oxidizing short-chain alkanes and carrying the canonical MCR-like protein genes. In addition, a consistent clustering pattern of methanogenic/methanotrophic archaea was found in the aCPSF1- and the 16S rRNA-phylogenies, in which candidatus order *Methanoliparales* was grouped with candidatus order *Methanophagales* (39) and candidatus genus *Syntrophoarchaeum*, which comprises propane- or butane-oxidizers (40). Therefore, aCPSF1 can be used in the phylogenetic analysis and taxonomy study of methanogenic and methanotrophic archaea.

### The aCPSF1 phylogeny groups the haloarchaea into the same three orders as those in the phylogenomic analysis.

Haloarchaea are distributed in high salinity environments and represent another most cultured archaeal group (18), whereas large number of species and genera in the 16S rRNA-based phylogenetic groups frequently have no recognizable phenotypic differences. Remarkably, multiple copies of the 16S rRNA gene, which usually show ∼5% sequence divergence, often occur in a single haloarchaeal species (41, 42), thereby adding difficulties in classification and identification of haloarchaea based on 16S rRNA homology (43, 44).

**FIG 5:**
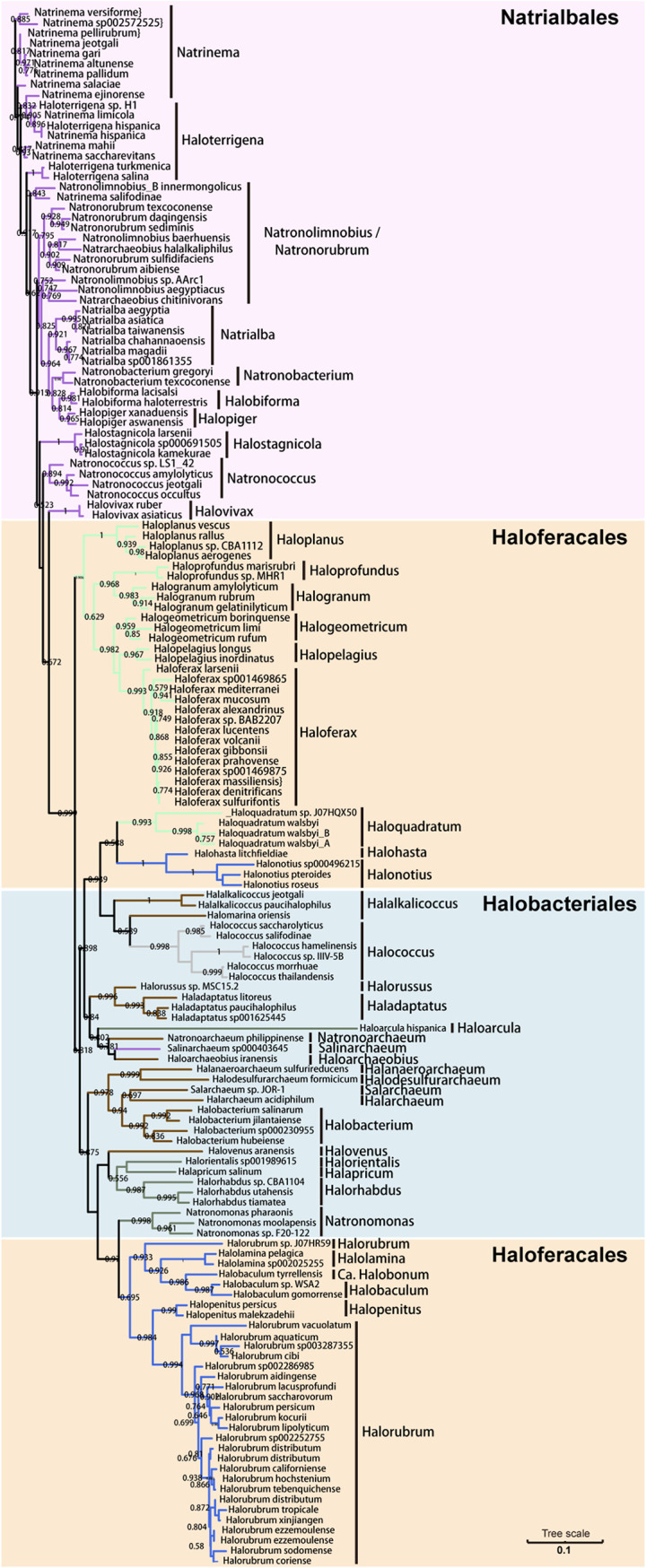
The aCPSF1 phylogeny of the class *Halobacteria*. The aCPSF1 orthologs from 152 Halobacteria genomes representing defined three orders of haloarchaea were used to construct a maximum likelihood phylogenetic tree. The trees were constructed based on a maximum likelihood (ML) analysis (IQ-TREE 1.6.12 in the LG+C20+R4+F model, 1,000 ultrafast bootstraps replicate), and visualized using iTOL v334. Bootstrap evaluation values of 1,000 iterations are indicated at branch nodes. Scale bar indicates number of substitutions per site.

To establish the aCPSF1 phylogeny of haloarchaea, we selected an aCPSF1 ortholog from each of the 152 haloarchaeal genomes (Dataset S1, “¤” marked). Phylogenetic analysis resulted in three major clades as orders of *Halobacteriales*, *Natrialbales*, and *Haloferacales*, however, the order *Haloferacales* was inserted by order *Halobacteriales* and split into two subgroups comprising of families *Halorubraceae* and *Haloferacaceae*, respectively (Fig. 5). This clustering pattern in general is similar to that of the haloarchaeal phylogenomic phylogeny based on the concatenation of 32 conserved proteins (18), except that the *Haloferacales* members were clustered as one clade. Like the phylogenomic analysis, the aCPSF1 phylogeny could better discriminate species in the order of *Halobacteriales* than that based on 16S rRNA. Thus, aCPSF1 could be used in the phylogenetic analysis and taxonomy study of haloarchaea as well.

Nevertheless, the aCPSF1 duplicates are also found in some haloarchaeal genomes and sequence similarities among the duplicates are between 60% to 100% (Dataset S4) that may cause misclassification or diversity overestimation, while this situation only occurs in 56 out of the 366 (15.3%) tested haloarchaeal genomes and primarily in *Halobacteriales* (Dataset S4).

### The aCPSF1-based taxonomy shows high resolution for lower archaeal taxa.

**FIG 6:**
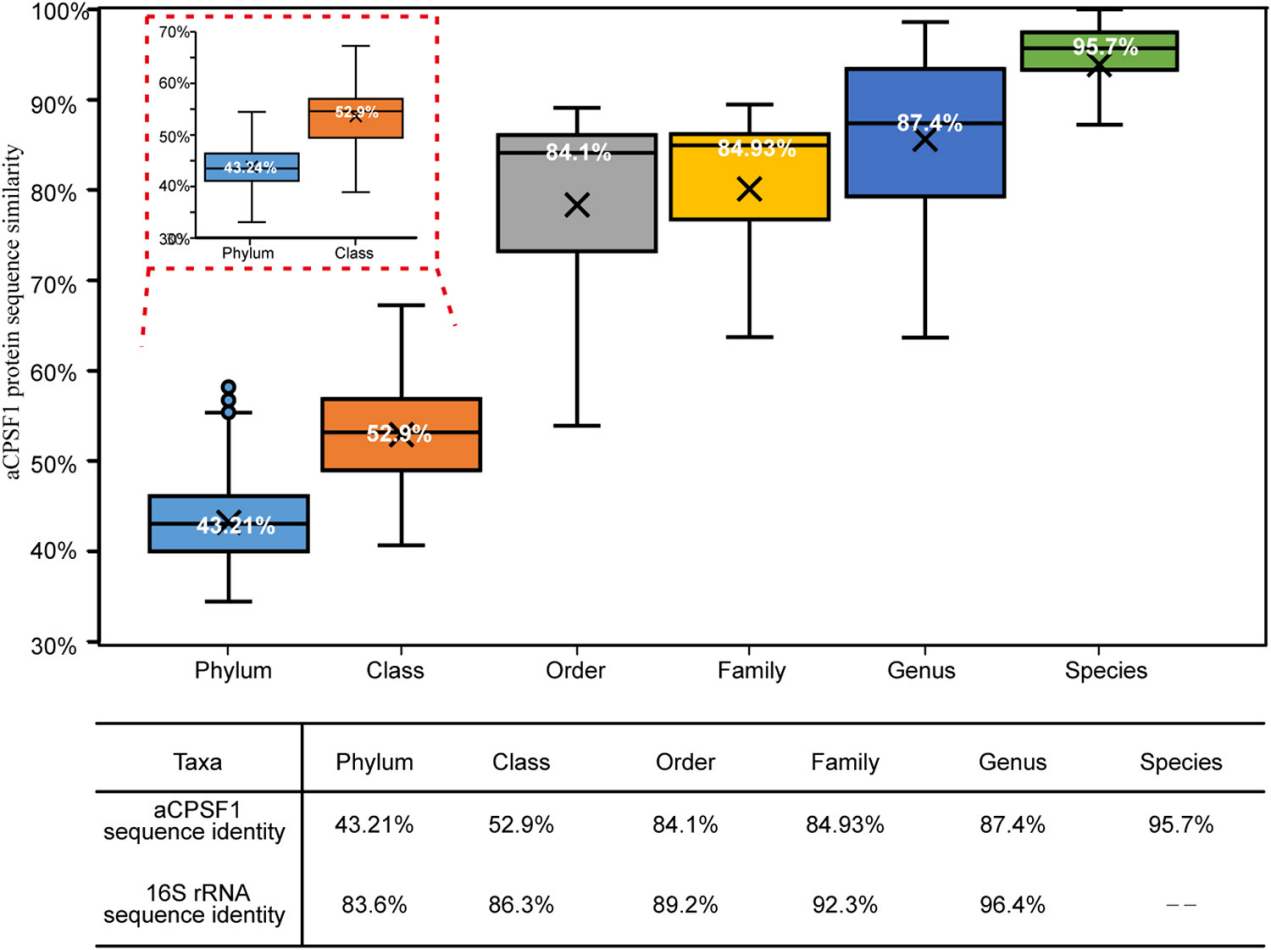
Rank normalization of the archaeal aCPSF1 taxonomy. In total, 779 aCPSF1 proteins were selected with each per archaeal genome/MAG (Dataset S2) that are deposited in NCBI and GTBD databases and have the identified taxonomic ranks. Sequence alignment was performed on consensus amino acids, and sequences identities at various taxonomic ranks were calculated. Box-plot diagram (upper panel) shows sequence identity distributions of 261 aCPSF1 proteins mostly from the cultured archaeal strains, and the insert shows the identities of 779 proteins at phylum and class levels. A table (lower panel) lists the median sequence identities of aCPSF1 proteins and the 16S rRNA genes (12) at various archaeal taxonomic ranks.

It is worth noting that the phylogenetic branches in aCPSF1 tree are primarily longer than those in a 16S rRNA tree (Fig. 3A and Fig. S2), suggesting that the aCPSF1 phylogeny could have a higher resolution in the identification of archaeal taxa. And considering the labor intensity of phylogenomic analysis in archaea classification, we attempted to establish an aCPSF1 taxonomic system for archaea. In total, each aCPSF1 ortholog was chosen from 779 genomes/MAGs (Dataset S2, “£” marked) that have been identified to the defined archaeal taxonomic ranks in NCBI and GTBD databases, in which 261 high-quality genomes (>98% completeness, Dataset S2, “£” marked) are from the cultured strains affiliating with four phyla: *Euryarchaeota*, *Crenarchaeota*, *Thaumarchaeota*, and *Thermoplasmatota*. By aligning the consensus amino acid sequences of the 779 aCPSF1 proteins and calculating the sequence identities at various taxonomic ranks, we were able to normalize the aCPSF1 identities at six archaeal taxonomic ranks. As shown in Fig. 6 and Dataset S2, our statistical analysis resulted in a distinguishable distribution of the aCPSF1 protein identities at six taxonomic ranks from species to phylum although dispersed identity values were found in the delineation of order, family, and genus, and poor hierarchical resolutions in family (84.9%) and order (84.1%).

Notably, the aCPSF1 taxonomy is particularly powerful in resolving archaeal species (95.7% identity) and genus (87.4% identity). The standard for species identification in aCPSF1 taxonomy is almost equivalent to that of the average nucleotide identity (ANI, 95%) circumscribing a species, and that for genus identification is more distinguishing than that of the 16S rRNA taxonomy (96.4% identity) (12). In addition, the aCPSF1-based taxonomy also shows a better differentiation on phylum (43.2%) and class (52.9%) of archaea than that of 16S rRNA taxonomy (phylum, 83.6%; class, 86.3%).

### Identifications of unclassified archaea by the aCPSF1 taxonomy system.

**FIG 7:**
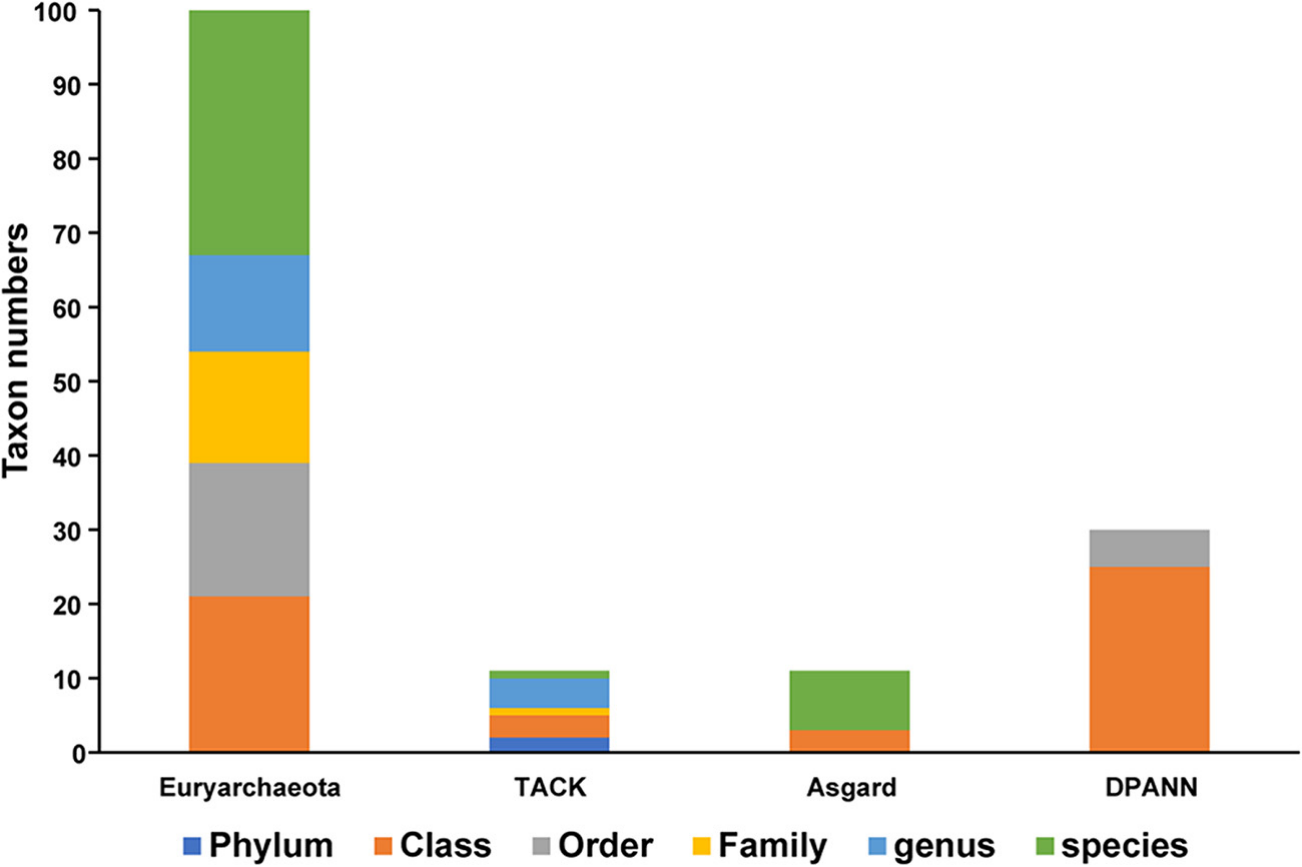
The taxonomic rank distributions of the aCPSF1 taxonomy identified 144 unclassed archaeal MAGs deposited in NCBI and GTDB. The 144 unclassed archaeal MAGs identified by aCPSF1 taxonomy are listed in Dataset S3.

We retrieved a total of 144 aCPSF1 orthologs from genomes/MAGs designated as “unclassified archaea” and “environmental samples” (Dataset S1 and S3) in NCBI database. These sequences were clustered in the defined archaeal clades at various sequence similarities (Fig. 2). Based on sequence identity of each aCPSF1 ortholog to the most closely related identified archaeal taxon in NCBI or GTDB, the 144 strains carrying the retrieved aCPSF1 were identified (Dataset S3) based on the normalized aCPSF1 sequence identity (%) standard (Fig. 6). The majority of the identified archaea fell in *Euryarchaeota*, and followed by DPANN (Fig. 7 and Dataset S3), and 43 genomes that were identified to species levels are primarily members of the class *Poseidoniia* of *Euryarchaeota* and candidatus phylum *Heimdallarchaeota* of Asgard archaea. Remarkably, the *Heimdallarchaeota* MAGs retrieved from different environments could be restricted just to one species. On the other hand, 55 genomes that are mainly from the superphylum DPANN could be only identified to class level, implying vast undiscovered species present in this archaeal superphylum with small genome size.

Identifications of some “unclassified archaea” using the aCPSF1 taxonomy are consistent with those obtained using the GTDB taxonomic system (Dataset S3). However, most of the “unclassified Euryarchaeota,” except for some *Methanomassiliicoccales*, in GTDB taxonomy could be identified to species by the aCPSF1 taxonomy. Therefore, the aCPSF1 taxonomy is a robust and practical system in surveying and identifying the archaeal diversity in environments.

### Using the aCPSF1 and 16S rRNA markers reveals similar archaeal diversities in samples from a South China Sea cold seep.

Given that more aCPSF1 orthologs than 16S rRNA gene are found in the archaea MAGs (Fig. 1a), and aCPSF1 taxonomy is more practical in identification of archaeal taxa than the 122 conserved proteins, we then tested if the aCPSF1-based taxonomy standard was applicable to survey archaeal diversity in a Southern China Sea (SCS) cold seep sediment. The cold seep sediments were sampled up to 180 cm below sea floor (bsf) using a piston core sampler, and sectioned to four according to the bsf depth (45). DNA extracted from each of the four sections was subjected to both 16S rRNA amplicon and metagenome sequencing.

**FIG 8:**
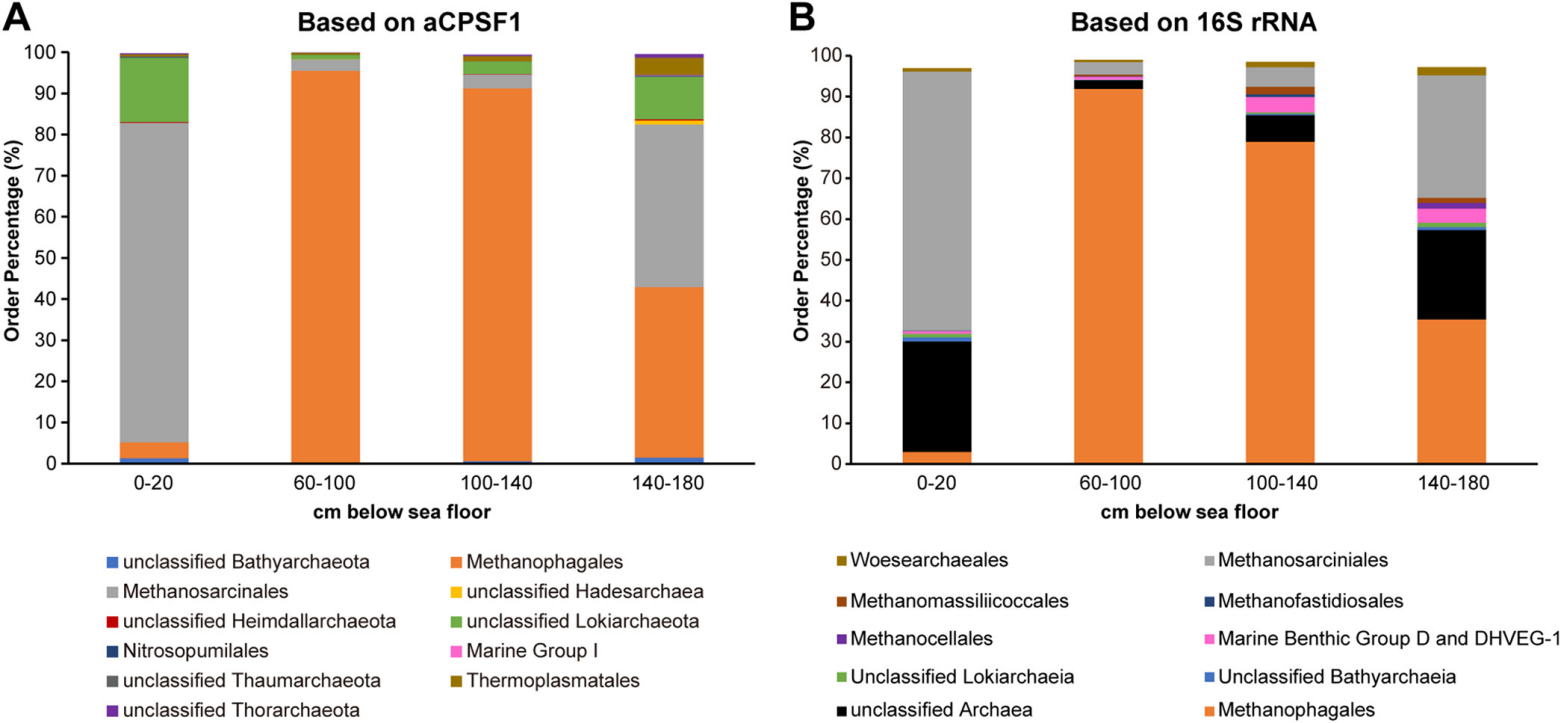
The aCPSF1 and 16S rRNA taxonomic systems surveyed similar archaeal diversities in a South China Sea cold seep sediment. The reductive sediment of a South China Sea cold seep was sampled until 180 cm below sea floor and sectioned into four, and then the total DNA was extracted. (A) Metagenome sequencing was performed on the DNA from each section, and the aCPSF1 orthologs were identified as described in Materials and Methods section. Archaea were identified to as lowest as possible taxonomic level based on the standard of aCPSF1 taxonomic ranks. (B) Archaeal 16S rRNAs diversity in each section was surveyed via high throughout amplicon sequencing on the sequence flanking the V4 and V5 regions, and archaea were identified according to the 16S rRNA sequence similarity.

In total 76,455 qualified 16S rRNA sequence reads were obtained from the four samples, and a rarefaction curve of OTUs identified by 16S rRNA homology plateaued (Fig. S3), indicating that the majority of the archaeal 16S rRNA genes were accessed. Meanwhile, through the metagenomic sequencing approach, we obtained 41-Mbp to 983-Mbp DNA sequences from the four samples. High-quality reads were mapped to the nonredundant aCPSF1genes, and archaeal diversity was estimated based on the aCPSF1 gene homology. Similarly, rarefaction curves of archaeal richness at both genus- and species-ranks identified by aCPSF1 homologs were also plateaued (Fig. S4), which showed about 30 to 40 species and 20 to less than 30 genera identified. While using 16S rRNA homology, more than 100 species identified in the sediment samples. This could be due to the higher identifying rates by construction of 16S rRNA libraries, or certain biases generated over-assessment. We found that archaea were mainly distributed in 60–140 cmbsf (Fig. 8A), and detected the representatives of archaeal superphyla of *Euryarchaeota*, TACK, and Asgard, except for DPANN. *Euryarchaeota* was the most predominant phylum in the four sediment sections, with the order *Methanosarcinales* and *Methanophagales* being most abundant from 0–20 and 140–180 cmbsf, and 60–140 cmbsf, respectively (Fig. 8A). *Lokiarchaeota*, *Heimdallarchaeota*, *Thorarchaeota*, *Thermoplasmatota*, *Thaumarchaeota*, and *Bathyarchaeota* were also detected. 16S rRNA gene surveyed the similar archaeal community structure (Fig. 8B) but additionally detected *Woesearchaeales* that affiliates with DPANN. Therefore, the archaeal community composition in the SCS cold seep surveyed by the aCPSF1-based taxonomy proposed in this study was similar to that by 16S rRNA-based taxonomy, supporting that aCPSF1 can be employed as a phylogenetic marker for archaea the taxonomy and diversity investigation study of archaea in environments.

In conclusion, our comparison of the aCPSF1 phylogeny with other widely used phylogenetic markers demonstrates that the highly conserved archaeal protein aCPSF1, the general transcription termination factor of archaea, may serve as an alternative phylogenetic marker for the classification and diversity survey of archaea in environments. It is nearly as powerful as phylogenomics using concatenated 122 conserved archaeal proteins in archaeal classification, but is significantly labor saving. Additionally, the aCPSF1 taxonomic system exhibits an excellent hierarchical resolution in delineation of archaeal genus and the species, which could compensate the lower resolution of 16S rRNA gene.

## MATERIALS AND METHODS

### Genome collection.

Available archaea genomes, proteins, and genes were downloaded from NCBI archaea database (ftp://ftp.ncbi.nlm.nih.gov/genomes/genbank/archaea/; February 2020). Taxonomy of archaea genomes were parsed by taxdump and NCBItax2lin (https://github.com/zyxue/ncbitax2lin) to complete NCBI lineages (superphylum, phylum, class, order, family, genus, and species). The completeness and contamination of archaea genomes was estimated using “checkm taxonomy_wf –genes” command in CheckM (v1.0.12) (21).

### Phylogenetic analyses of aCPSF1 orthologs, 122 marker proteins, 16S rRNA, and McrABG genes.

aCPSF1 orthologs were obtained by aligning the archaeal proteins to hidden Markov models (HMMs) of beta-CASP RNase with a suggested bit score of 580 (TIGR03675), using hmmsearch (30). The resulting aCPSF1 orthologs, with one per aligned genome, were multi-aligned by the program MAFFT 7.455 (46) using “–auto” algorithm, followed by sequence trimming using trimAl 1.4.1 in default parameters (47). Maximum likelihood (ML) phylogenetic trees were inferred with FastTree v.2.1.10 under the WAG+GAMMA model (48).

One-hundred and 22 archaeon-specific protein markers were identified using HMMs (19, 37), aligned individually using hmmalign with default parameters (49, 50). The 122 markers concatenated alignment was trimmed using BMGE with flags “-t AA -m BLOSUM30” (51), followed by maximum likelihood phylogenies in IQ-TREE (v.1.6.12) with “LG+I+G4” mode and 1,000 times ultrafast bootstrapping (52).

Available 16S rRNA genes (>1,200 bp) of each genome were retrieved from above downloaded gene sequences files, and aligned using SINA (v.1.7.1) with default parameters (53).The 16S rRNA gene sequences maximum-likelihood tree was built by IQ-TREE (v.1.6.12) with “GTR+I+G4” model and option of “-bb 1000”.

Three HMMs of TIGR03256, TIGR03257, and TIGR03259 were used for McrABG orthologs screening from the above downloaded proteins, using hmmsearch with bit scores of 768, 516, and 172, respectively. The three sets of Mcr proteins were multi-aligned individually by the program MAFFT 7.455 (46) using “–auto” algorithm. The resulting McrABG concatenated alignment was used for ML phylogenetic tree construction with FastTree v.2.1.10 under the WAG+GAMMA model (48). All phylogenic trees were visualized with iTOL v3 (https://itol.embl.de/).

### Rank normalization of the aCPSF1 taxonomy.

Two-hundred and 61 high-quality genomes from cultured isolates with >98% completeness and defined taxonomic affiliation at species levels (Dataset S2, “Ł” marked) were selected first for taxonomic rank normalization using aCPSF1 identity metric, which was produced from SIAS (sequence identity and similarity; http://imed.med.ucm.es/Tools/sias.html). After removed, those genomes/MAGs named “unclassified archaea” or “environmental samples,” we manually selected 779 genomes in total from all archaeal phyla (Dataset S2, “£” marked), including those can only be identified to phylum or class levels, to validate the broad applicability of aCPSF1 identity metric across all archaeal taxa.

### Sample collection from a South China Sea cold seep sediment.

The reductive cold seep sediments in 1,165-m depth of seawater in the active site of Formosa Ridge cold seep (22°06'89.05"N; 119°17'16.384"E) were sampled up to 180 cm bsf using remote operated vehicle (ROV) and piston core sampler during the KEXUE-2019 expedition. Sampled sediment column was sectioned into four sections, and immediately transferred to a sterile plastic bag, and stored at –80°C until use.

### 16S rRNA sequencing of archaea.

Total DNA was extracted from each section of the sediment columns. Archaeal 16S rRNAs diversity in each section was surveyed via high throughout sequencing. Primers Arch519F and Arch915R were used to amplify a 399-bp fragment of the archaeal 16S rRNA gene flanking the V4 and V5 regions. Purified amplicons were sequenced by Novogene company (Beijing, China) and processed the Illumina Miseq sequencing with standard protocols and the data using QIIME (version 1.7.0) pipeline. Operational taxonomic units (OTU) tables were generated from the pipeline.

### Metagenome sequencing.

Total DNA was extracted from the four sections of reduced sediment (∼0.5 g) using the MoBio Powersoil DNA isolation kit (MoBio, Carlsbad, CA, USA) according to the manufacturer’s protocol, and was then paired-end sequenced on Illumina HiSeq TM2000 platform by Novogene company (Beijing, China). The raw paired-end metagenomic sequencing reads of two repeat samples were filtered and quality-controlled first using read_qc module in metaWRAP.

### Mapping aCPSF1 orthologs in cold seep sediment.

The above available aCPSF1 genes were first clustered using CD-HIT-EST (54) at 100% identity and 100% coverage to reduce redundancy. High-quality reads were mapped to the nonredundant gene sets. Using Bowtie2 V.2.2.4 (55) with an end-to-end alignment to calculate their relative abundances, the taxonomic profiling of aCPSF1 genes was used to assess the archaeal diversity in cold-seep sediment samples as described previously (56).

### Data availability.

The metagenome sequencing raw data have been deposited in NCBI Short Read Archive under BioProject accession number of PRJNA722826 (https://www.ncbi.nlm.nih.gov/bioproject/PRJNA722826). The Illumina sequencing data of archaeal and bacteria 16S rRNA gene V4 and V5 regions amplified from the sediment samples were deposited in the NCBI Short Read Archive under accession number PRJNA724900 (https://dataview.ncbi.nlm.nih.gov/object/PRJNA724900?reviewer=vf0ou2uj6a1kfhaieek146bhus). All the amino acid and nucleotide sequences of the 2,026 a CPSF1 orthologs that we have retrieved from NCBI in this study have been uploaded and can be accessed publicly through the weblink of ftp://download.nmdc.cn/attachment/aCPSF1/ with IE explorer or ftp software.
