# Supplementary material for: The Archaeal Transcription Termination Factor aCPSF1 is a Robust Phylogenetic Marker for Archaeal Taxonomy
Source: Microbiol Spectr. 2021 Dec 8;9(3):e01539-21. doi: 10.1128/spectrum.01539-21 (PMC8653824; doi:10.1128/spectrum.01539-21)
Supplement: SUPPLEMENTAL FILE 1 — Supplemental material. Download SPECTRUM01539-21_Supp_1_seq4.pdf, PDF file, 5.6 MB [file spectrum01539-21_supp_1_seq4.pdf]

Supporting Information for

The archaeal transcription termination factor aCPSF1 is a robust phylogenetic marker for archaeal taxonomy

This file contains:

Supplementary Figures. S1 to S4;

Caption for Supplementary Dataset S1 Information of the investigated archaeal genomes downloaded from NCBI database

Caption for Supplementary Dataset S2 Information of the 779 archaeal genomes used for taxonomy ranking based on aCPSF1

Caption for Supplementary Dataset S3 Identified 144 unclassified archaeal genomes in NCBI database based on aCPSF1 taxonomy system

Caption for Supplementary Dataset S4 Sequence similarities of the duplicated aCPSF1 orthologs in 56 out of 366 haloarchaeal genomes

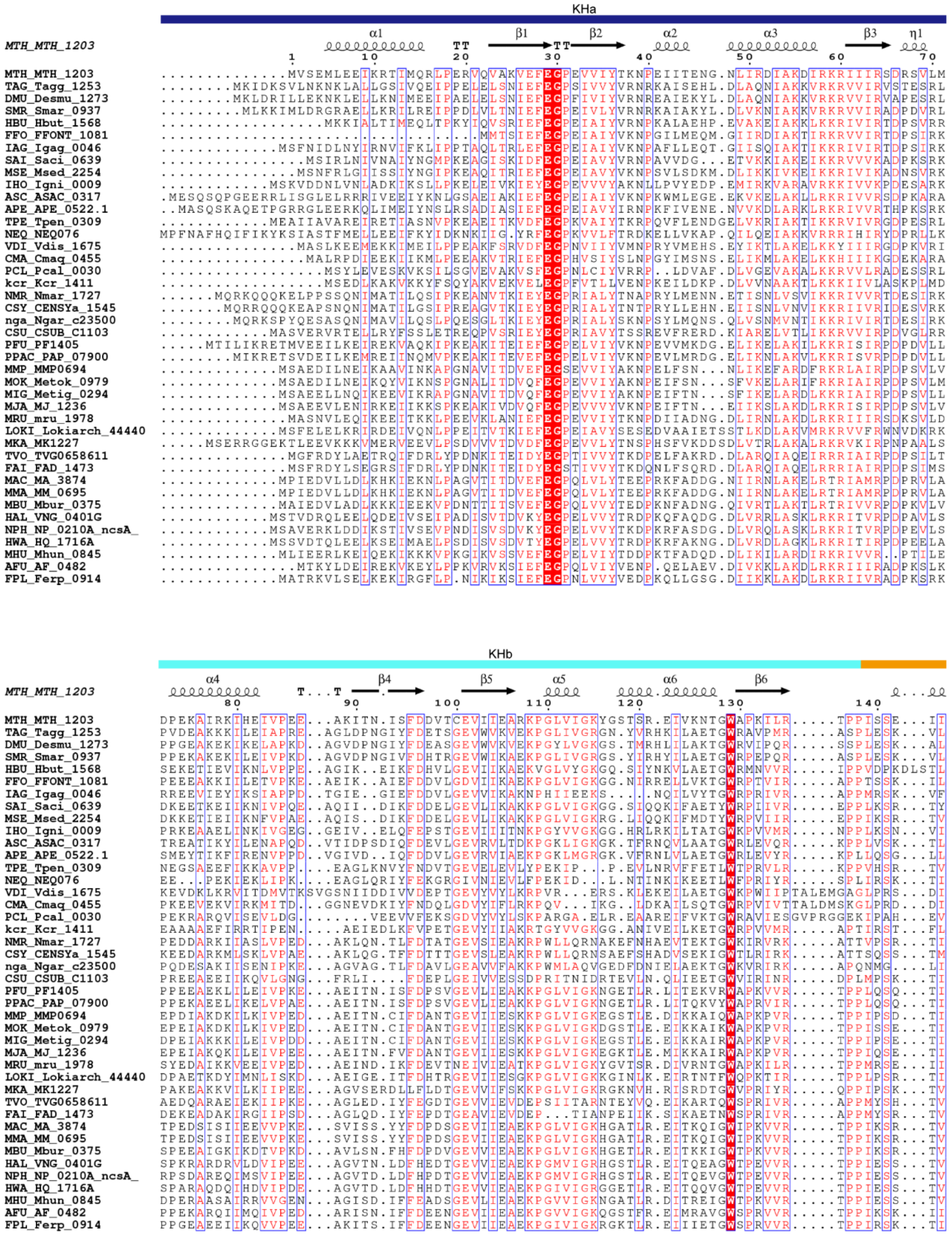

KHa
MTH_MTH_1203
α1
β1
β2
α2
α3
β3
η1
MTH_MTH_1203
TAG_Tagg_1253
DMU_Desmu_1273
SMR_Smar_0937
HBU_Hbut_1568
FFO_FFONT_1081
IAG_Igag_0046
SAI_Saci_0639
MSE_Msed_2254
IHO_Igni_0009
ASC_ASAC_0317
APE_APE_0522.1
TPE_Tpen_0309
NEQ_NEQ076
VDI_Vdis_1675
CMA_Cmaq_0455
PCL_Pcal_0030
kcr_Kcr_1411
NMR_Nmar_1727
CSY_CENSYa_1545
nga_Ngar_c23500
CSU_CSUB_C1103
PFU_PF1405
PPAC_PAP_07900
MMP_MMP0694
MOK_Metok_0979
MIG_Metig_0294
MJA_MJ_1236
MRU_mru_1978
LOKI_Lokiarch_44440
MKA_MK1227
TVO_TVG0658611
FAI_FAD_1473
MAC_MA_3874
MMA_MM_0695
MBU_Mbur_0375
HAL_VNG_0401G
NPH_NP_0210A_ncsA_
HWA_HQ_1716A
MHU_Mhun_0845
AFU_AF_0482
FPL_Ferp_0914
KHb
MTH_MTH_1203
α4
β4
β5
α5
α6
β6

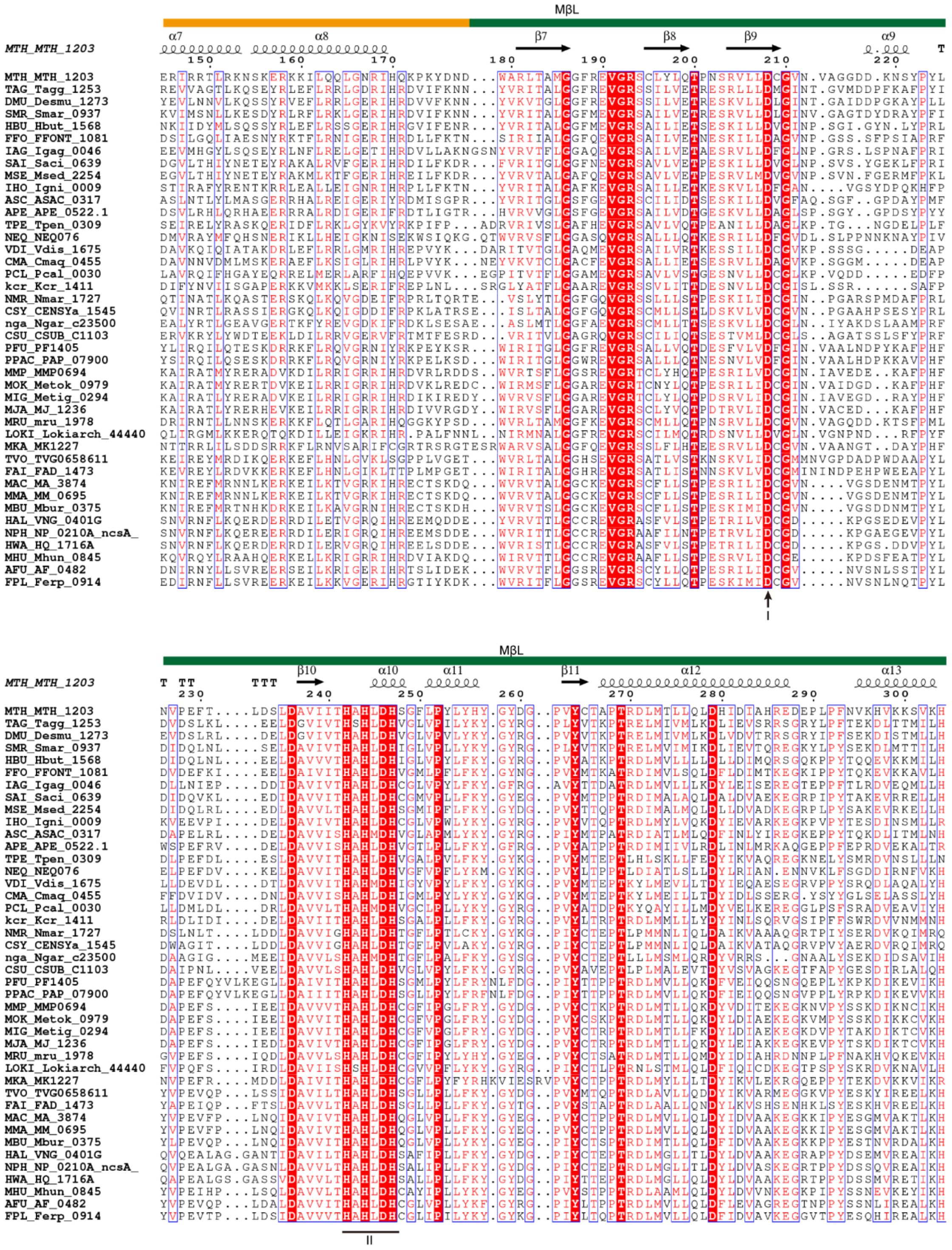
MβL
MTH_MTH_1203
α7 α8 β7 β8 β9 α9
150 160 170 180 190 200 210 220
MTH_MTH_1203
TAG_Tagg_1253
DMU_Desmu_1273
SMR_Smar_0937
HBU_Hbut_1568
FFO_FFONT_1081
IAG_Igag_0046
SAI_Saci_0639
MSE_Msed_2254
IHO_Igni_0009
ASC_ASAC_0317
APE_APE_0522.1
TPE_Tpen_0309
NEQ_NEQ076
VDI_Vdis_1675
CMA_Cmaq_0455
PCL_Pcal_0030
kcr_Kcr_1411
NMR_Nmar_1727
CSY_CENSYa_1545
nga_Ngar_c23500
CSU_CSUB_C1103
PFU_PF1405
PPAC_PAP_07900
MMP_MMP0694
MOK_Metok_0979
MIG_Metig_0294
MJA_MJ_1236
MRU_mru_1978
LOKI_Lokiarch_44440
MKA_MK1227
TVO_TVG0658611
FAI_FAD_1473
MAC_MA_3874
MMA_MM_0695
MBU_Mbur_0375
HAL_VNG_0401G
NPH_NP_0210A_ncsA_
HWA_HQ_1716A
MHU_Mhun_0845
AFU_AF_0482
FPL_Ferp_0914
I
MβL
MTH_MTH_1203
β10 α10 α11 β11 α12 α13
230 240 250 260 270 280 290 300
II

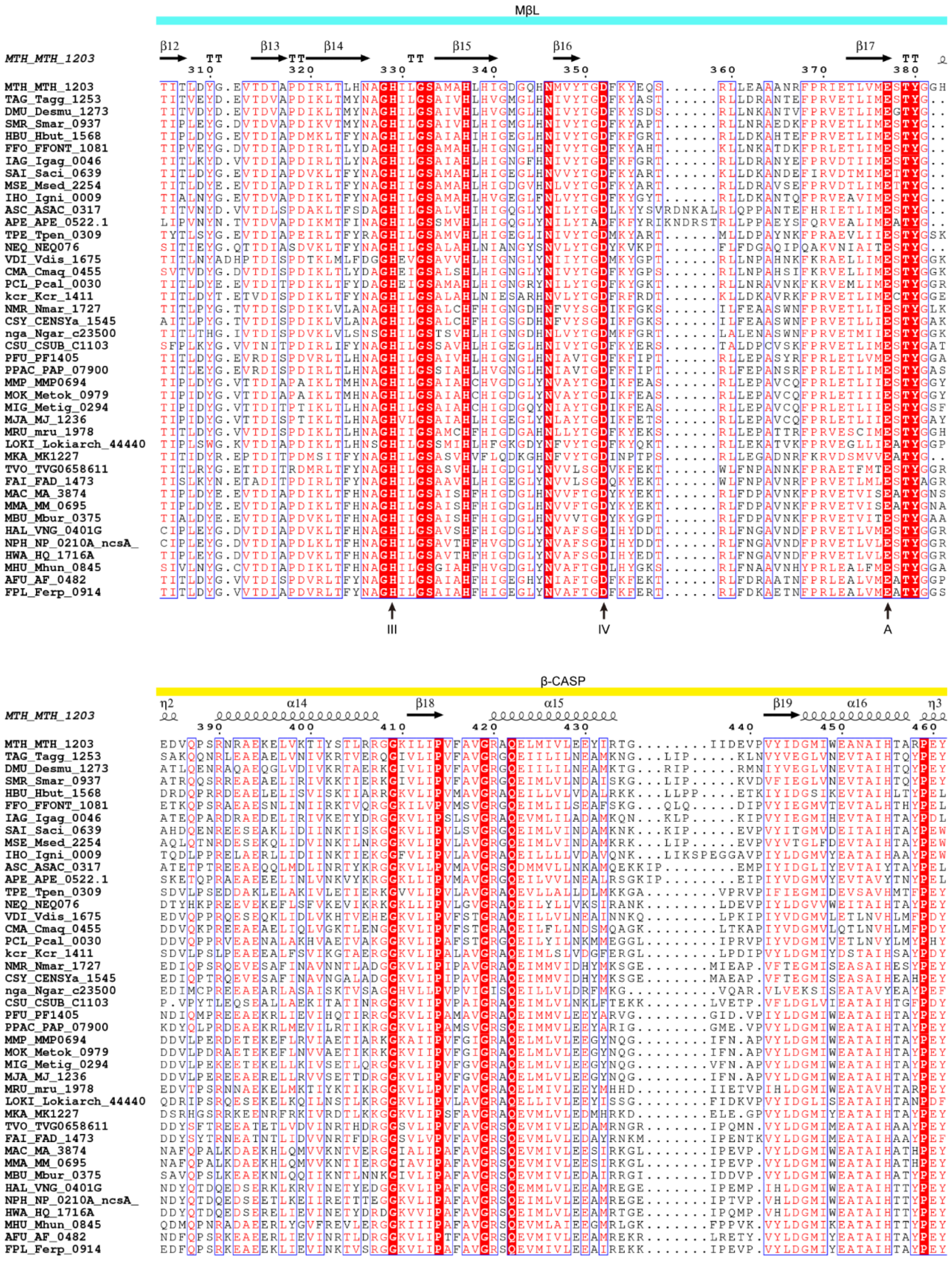

MβL
MTH_MTH_1203
β12 TT β13 TT β14 TT β15 β16 β17 TT
310 320 330 340 350 360 370 380
MTH_MTH_1203
TAG_Tagg_1253
DMU_Desmu_1273
SMR_Smar_0937
HBU_Hbut_1568
FFO_FFONT_1081
IAG_Igag_0046
SAI_Saci_0639
MSE_Msed_2254
IHO_Igni_0009
ASC_ASAC_0317
APE_APE_0522.1
TPE_Tpen_0309
NEQ_NEQ076
VDI_Vdis_1675
CMA_Cmaq_0455
PCL_Pcal_0030
kcr_Kcr_1411
NMR_Nmar_1727
CSY_CENSYa_1545
nga_Ngar_c23500
CSU_CSUB_C1103
PFU_PF1405
PPAC_PAP_07900
MMP_MMP0694
MOK_Metok_0979
MIG_Metig_0294
MJA_MJ_1236
MRU_mru_1978
LOKI_Lokiarch_44440
MKA_MK1227
TVO_TVG0658611
FAI_FAD_1473
MAC_MA_3874
MMA_MM_0695
MBU_Mbur_0375
HAL_VNG_0401G
NPH_NP_0210A_ncsA_
HWA_HQ_1716A
MHU_Mhun_0845
AFU_AF_0482
FPL_Ferp_0914
III
IV
A
β-CASP
MTH_MTH_1203
η2 α14 β18 α15 β19 α16 η3
390 400 410 420 430 440 450 460
MTH_MTH_1203
TAG_Tagg_1253
DMU_Desmu_1273
SMR_Smar_0937
HBU_Hbut_1568
FFO_FFONT_1081
IAG_Igag_0046
SAI_Saci_0639
MSE_Msed_2254
IHO_Igni_0009
ASC_ASAC_0317
APE_APE_0522.1
TPE_Tpen_0309
NEQ_NEQ076
VDI_Vdis_1675
CMA_Cmaq_0455
PCL_Pcal_0030
kcr_Kcr_1411
NMR_Nmar_1727
CSY_CENSYa_1545
nga_Ngar_c23500
CSU_CSUB_C1103
PFU_PF1405
PPAC_PAP_07900
MMP_MMP0694
MOK_Metok_0979
MIG_Metig_0294
MJA_MJ_1236
MRU_mru_1978
LOKI_Lokiarch_44440
MKA_MK1227
TVO_TVG0658611
FAI_FAD_1473
MAC_MA_3874
MMA_MM_0695
MBU_Mbur_0375
HAL_VNG_0401G
NPH_NP_0210A_ncsA_
HWA_HQ_1716A
MHU_Mhun_0845
AFU_AF_0482
FPL_Ferp_0914

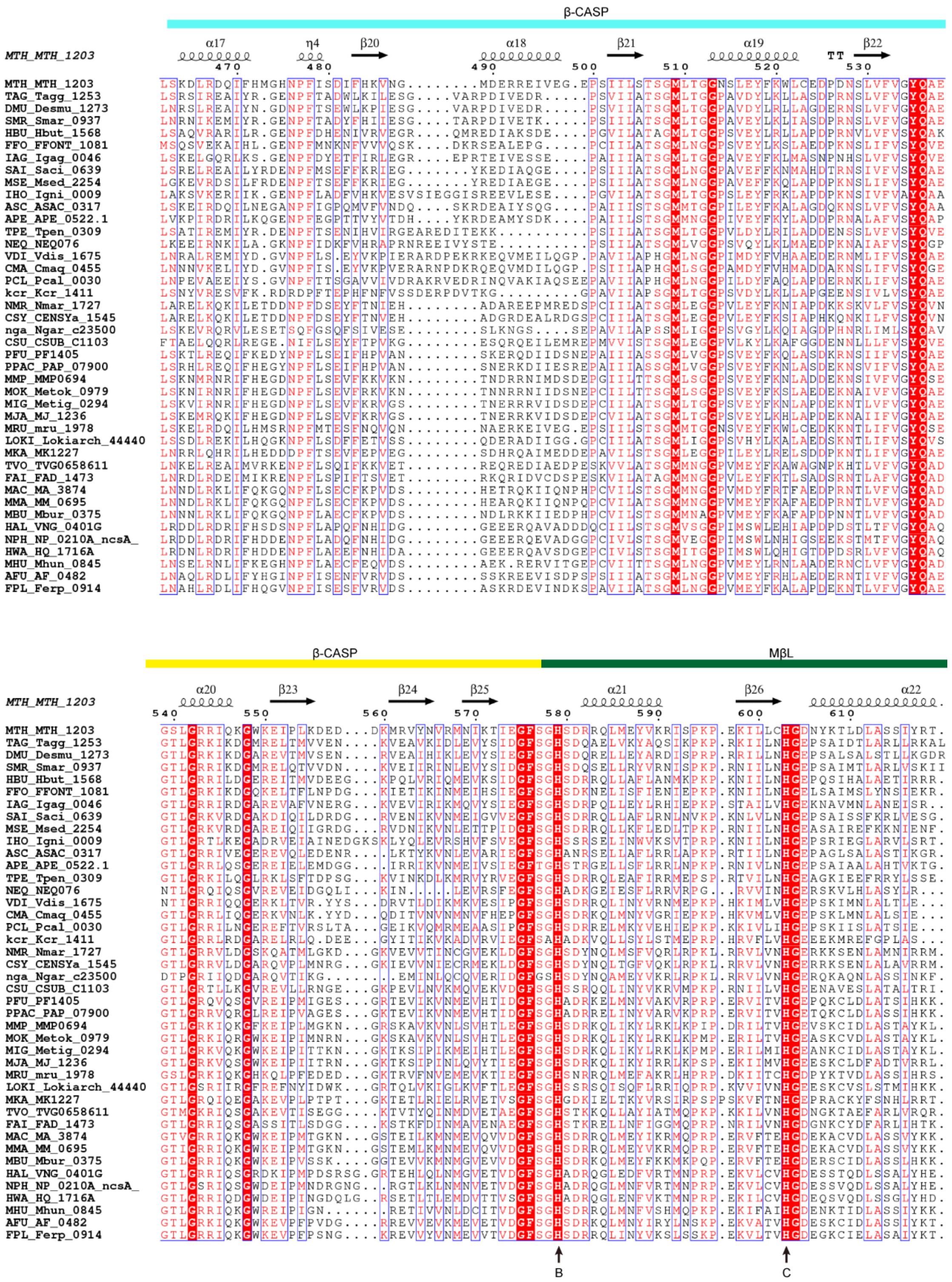
β-CASP
MTH_MTH_1203
α17
η4
β20
α18
β21
α19
TT
β22
β-CASP
MβL
α20
β23
β24
β25
α21
β26
α22
B
C

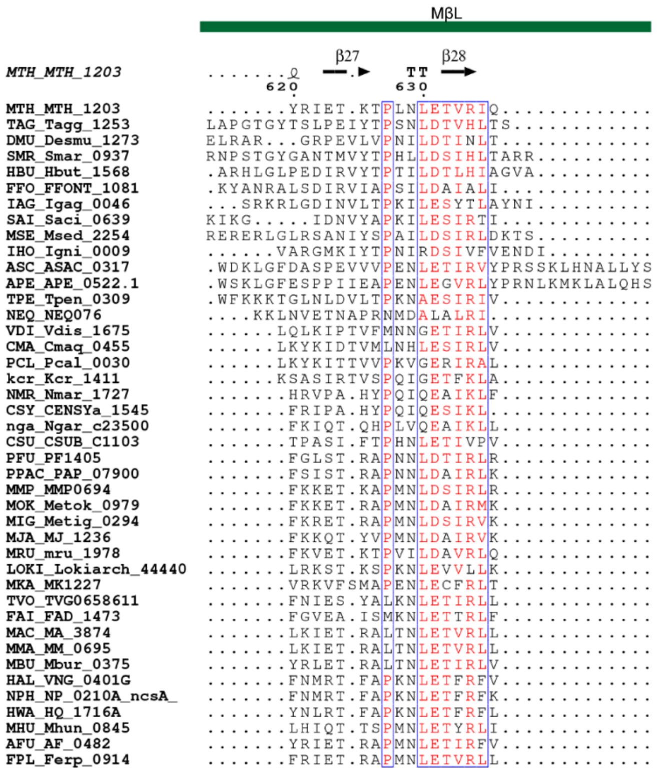

Figure S1. Sequence alignment, secondary structure, and functional motifs of the aCPSF1 orthologs. Sequences were aligned using ClustalW program (1) and the diagram was prepared using ESPript program (2). Identical residues are highlighted as white type on a red background and similar residues are shown as red type. Species: MTH, *Methanothermobacter thermautotrophicus*; TAG, *Thermosphaera aggregans*; DMU, *Desulfurococcus mucosus*; SMR, *Staphylothermus marinus*; HBU, *Hyperthermus butylicus*; FFO, *Fervidicoccus fontis*; IAG, *Ignisphaera aggregans*; SAI, *Sulfolobus acidocaldarius* DSM 639; MSE, *Metallosphaera sedula*; IHO, *Ignicoccus hospitalis*; ASC, *Acidilobus saccharovorans*; APE, *Aeropyrum pernix*; TPE, *Thermofilum pendens*; NEQ, *Nanoarchaeum equitans*; VDI, *Vulcanisaeta distributa* ; CMA, *Caldivirga maquilingensis*; PCL, *Pyrobaculum calidifontis*; Kcr, Ca. *Korarchaeum cryptofilum*; NMR, *Nitrosopumilus maritimus*; CSY, *Cenarchaeum symbiosum*; nga, Ca. *Nitrososphaera gargensis*; CSU, Ca. *Caldiarchaeum*

subterraneum; PFU, *Pyrococcus furiosus* DSM 3638 ; PPAC, *Palaeococcus pacificus*; MMP, *Methanococcus maripaludis* S2; MOK, *Methanothermococcus okinawensis*; MIG, *Methanotorris igneus*; MJA, *Methanocaldococcus jannaschii*; MRU, *Methanobrevibacter ruminantium*; HAL, *Halobacterium salinarum* NRC-1; NPH, *Natronomonas pharaonis*; HWA, *Haloquadratum walsbyi* DSM 16790; MHU, *Methanospirillum hungatei*; AFU, *Archaeoglobus fulgidus* DSM 4304; FPL, *Ferroglobus placidus*. Secondary structural elements and the domains of the aCPSF1 ortholog (MTH1203) from *M. thermautotrophicus* are shown at the sequence top. The seven conserved motifs, Motifs I-IV and Motifs A–C, which are featured in the metallo-β-lactamase (MβL) superfamily proteins are labeled beneath the consensus residues.

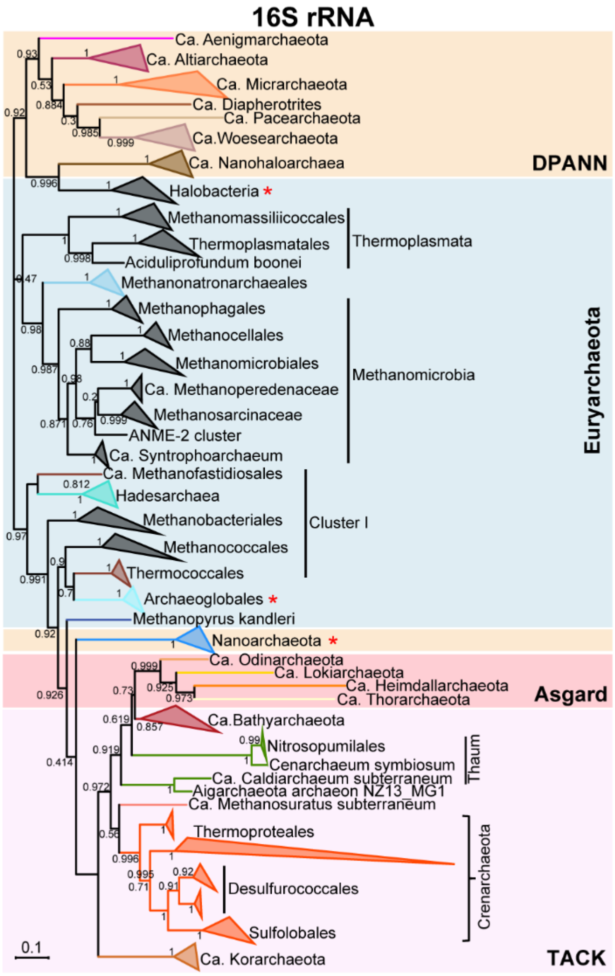

Figure S2. **The 16S rRNA phylogeny of archaea in comparison of the aCPSF1 phylogeny shown in Figure 3A.** 16S rRNA genes (>1200 bp) were retrieved from the same 143 representative archaeal genomes used in Figure 3A. Consensus 1200 bp-nucleotides of the 16S rRNA genes were used to construct a maximum likelihood phylogenetic trees using IQ-TREE (v.1.6.12) with "LG+I+G4" mode and 1000 times ultrafast. The phylogenetic tree was visualized with iTOL v5 (https://itol.embl.de/). Branch support values are indicated by percentages. Scale bar indicates the number of substitutions per site.

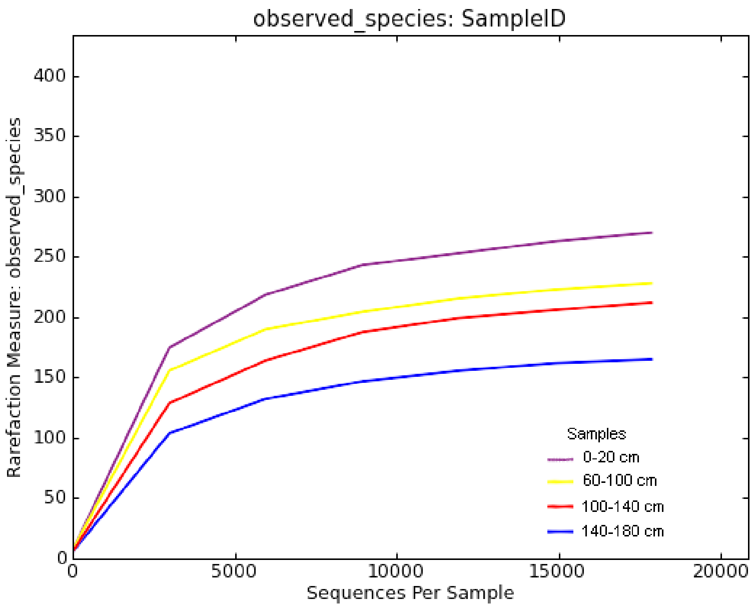

Figure S3. Rarefaction curves of the archaeal OTUs against 16S rRNA sequence reads in the four cold seep samples from the South China Sea. Duplicate 16S rRNA amplicon sequencings were performed for each sample and OTUs were clustered based on the threshold of 97% sequence similarity of 16S rRNA sequence.

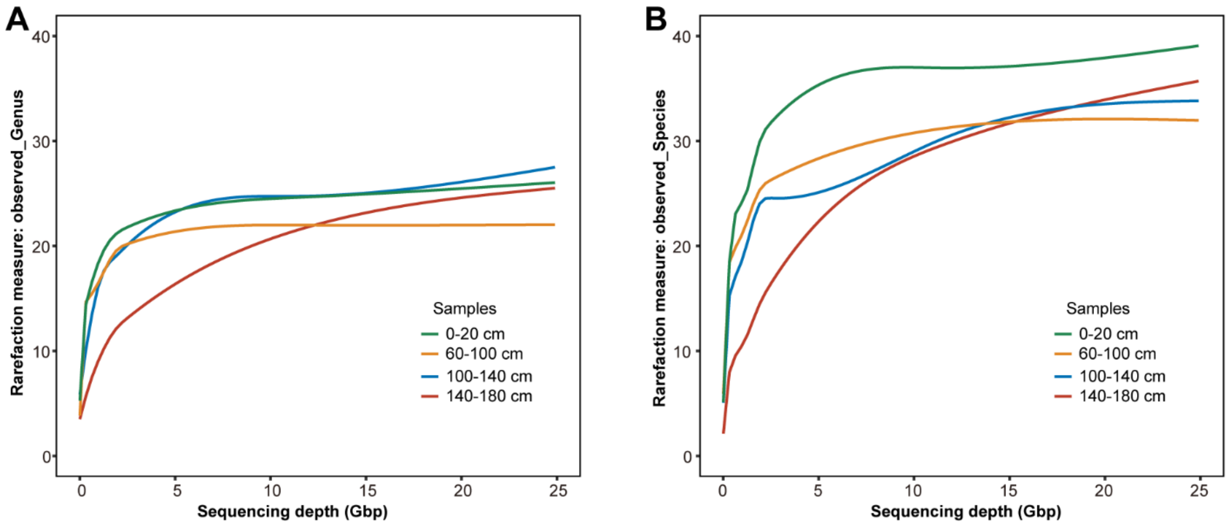

Figure S4. Rarefaction curves of archaeal richness at genus (A) and species (B) levels based on abundance of aCPSF1 genes in different sequencing depths. Rarefaction tool kit (RTK) (3) was used to rarefy a series of sequencing depths, *e.g.*, 5, 10 and 15Gb, and estimate the archaeal alpha-richness. aCPSF1 mapped reads were retrieved from the metagenomic sequences of each sample and the archaeal richness were merged based on the taxonomy of aCPSF1 genes listed in Supplementary Dataset S1.

SI References

1. Larkin MA, Blackshields G, Brown NP, Chenna R, McGettigan PA, McWilliam H, Valentin F, Wallace IM, Wilm A, Lopez R, Thompson JD, Gibson TJ, Higgins DG. 2007. Clustal W and Clustal X version 2.0. Bioinformatics 23:2947-8.
2. Gouet P, Courcelle E, Stuart DI, Metoz F. 1999. ESPript: analysis of multiple sequence alignments in PostScript. Bioinformatics 15:305-8.

3 Saary, P, Forslund, K, Bork, P and Hildebrand, F. 2017. RTK: efficient rarefaction analysis of large datasets. Bioinformatics 33(16): 2594-2595
